# Early referring saved lives in kidney transplant recipients with COVID-19: a beneficial role of telemedicine

**DOI:** 10.3389/fmed.2023.1252822

**Published:** 2023-09-19

**Authors:** Ivan Zahradka, Vojtech Petr, Katarina Jakubov, Istvan Modos, Filip Hruby, Ondrej Viklicky

**Affiliations:** ^1^Department of Nephrology, Institute for Clinical and Experimental Medicine, Prague, Czechia; ^2^Department of Information Technology, Institute for Clinical and Experimental Medicine, Prague, Czechia; ^3^Transplant Laboratory, Institute for Clinical and Experimental Medicine, Prague, Czechia

**Keywords:** COVID-19, SARS-CoV-2, telehealth, kidney transplantation, outcomes, mortality

## Abstract

**Introduction:**

There is a strong impetus for the use of telemedicine for boosting early detection rates and enabling early treatment and remote monitoring of COVID-19 cases, particularly in chronically ill patients such as kidney transplant recipients (KTRs). However, data regarding the effectiveness of this practice are lacking.

**Methods:**

In this retrospective, observational study with prospective data gathering we analyzed the outcomes of all confirmed COVID-19 cases (*n* = 955) in KTRs followed at our center between March 1, 2020, and April 30, 2022. Risk factors of COVID-19 related mortality were analyzed with focus on the role of early referral to the transplant center, which enabled early initiation of treatment and remote outpatient management. This proactive approach was dependent on the establishment and use of a telemedicine system, which facilitated patient-physician communication and expedited diagnostics and treatment. The main exposure evaluated was early referral of KTRs to the transplantation center after confirmed or suspected COVID-19 infection. The primary outcome was the association of early referral to the transplantation center with the risk of death within 30 days following a COVID-19 diagnosis, evaluated by logistic regression.

**Results:**

We found that KTRs who referred their illness to the transplant center late had a higher 30-day mortality (4.5 vs. 13.6%, *p* < 0.001). Thirty days mortality after the diagnosis of COVID-19 was independently associated with late referral to the transplant center (OR 2.08, 95% CI 1.08–3.98, p = 0.027), higher age (OR 1.09, 95% CI 1.05–1.13, *p* < 0.001), higher body mass index (OR 1.06, 95% CI 1.01–1.12, *p* = 0.03), and lower eGFR (OR 0.96, 95% CI 0.94–0.98, *p* < 0.001) in multivariable logistic regression. Furthermore, KTRs who contacted the transplant center late were older, had longer time from transplantation, lived farther from the center and presented with higher Charlson comorbidity index.

**Discussion:**

A well-organized telemedicine program can help to protect KTRs during an infectious disease outbreak by facilitating pro-active management and close surveillance. Furthermore, these results can be likely extrapolated to other vulnerable populations, such as patients with chronic kidney disease, diabetes or autoimmune diseases requiring the use of immunosuppression.

## 1. Introduction

The COVID-19 pandemic is one of the most urgent global medical problems of recent decades. Kidney transplant recipients (KTRs) represent one of the most vulnerable patient populations (1) due to the combination of various factors such as chronic kidney disease (2), immunosuppression (2), frequent comorbidities, and socio-economic status (3).

Clinical outcomes for both the general population (4) and KTRs (5) have recently greatly improved due to vaccination programs, new treatment options, and the evolution of the virus towards less pathogenic strains (6). However, novel variants, such as the Omicron variant, remain highly contagious and the case fatality rate is still reported to be around 2% for KTRs (5). Furthermore, the potentiality of emergence of new, more dangerous SARS-CoV-2 variants as well as another pandemic outbreak in the globalized world (7) should not be underestimated. Therefore, surveillance and pro-active management are still crucial in order to minimize preventable complications of COVID-19.

Significant challenges in access to healthcare still exist, even in countries with universal healthcare systems. Among these are living in remote locations, age, patient comorbidities, lower socioeconomic status, and impaired mobility or cognitive functions. Telehealth can help overcome many of these challenges by enabling closer patient-physician communication even across distance. Indeed, many complications in KTRs are prevented with early detection (8) and treatment, therefore thoughtful telehealth shows great promise especially with the myriad challenges that arise in transplant medicine (8, 9). However, effective support and integration of telehealth is a challenge not yet fully delineated, and it is unknown to what extent remote measures directly affect clinical outcomes.

This study reports the clinical outcomes of a large KTR cohort at a regional referral transplant center during the COVID-19 pandemic in the Czech Republic. The main focus was to assess the role of early implementation of telehealth in COVID-related patient outcomes.

## 2. Methods

**Figure 1:**
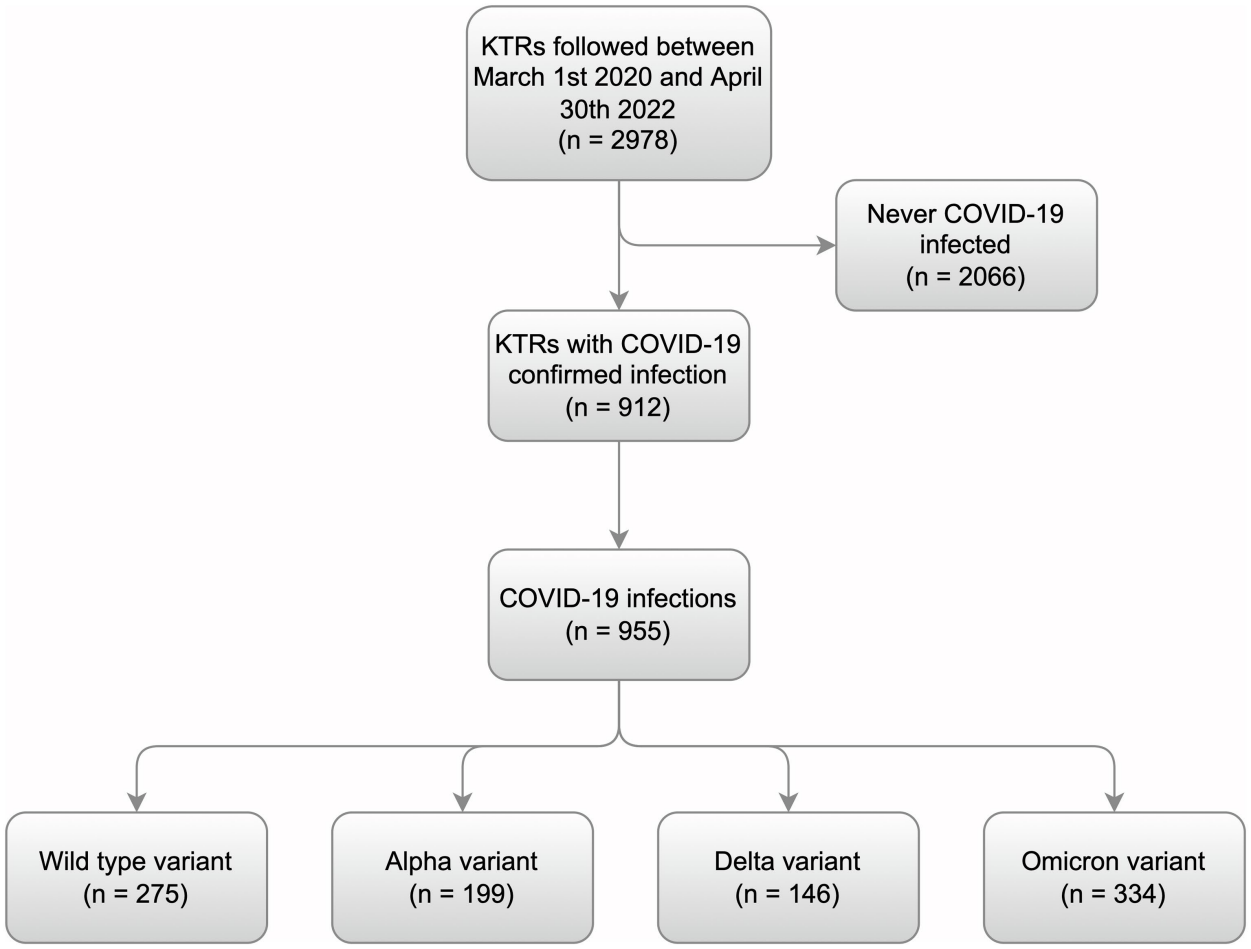
Study flow-chart. All individual cases of COVID-19 in the kidney transplant recipients followed at our transplantation center between March 1, 2020, and April 30, 2022, were identified. The cases were divided into individual periods depending on the presumed dominant virus variant at the time. The wild-type period began with the first case (March 1, 2020) and continued up to January 31, 2021. The Alpha period lasted between February 1, 2021, and September 30, 2021. The Delta period followed between October 1, 2021, and December 31, 2021. The Omicron period started on January 1, 2022.

This is a single center retrospective observational cohort study with prospective data gathering. We included 955 COVID-19 cases in all the 912 infected KTRs followed at our center between March 1, 2020 (first confirmed COVID-19 case in the Czech Republic) and April 30, 2022 (the date when the analysis was started). Study flow-chart is depicted in Figure 1.

The main research question was to assess the effect of our telemedicine guided approach towards early diagnostics and management of COVID-19 cases in KTRs on COVID-19 associated mortality in the context of other risk factors. The main exposure was early referral of KTRs to the transplantation center after confirmed or suspected COVID-19 infection. The primary outcome was the association of early referral with the risk of death within 30 days following a COVID-19 diagnosis.

The secondary aim was to describe the impact of the COVID-19 pandemic on KTRs in the Czech Republic.

### 2.1. Telehealth and outpatient management

Telehealth is broadly defined as the use of electronic information and communications technologies to provide and support health care when distance separates the participants (10). Telehealth may include simple methods such as a telephone call and sophisticated methods such as smartphone applications (11).

For the management of the COVID-19 cases and for this study, we defined telehealth as any method used for the purpose of remote contact between the patient, family members, or other healthcare providers with the transplant center. Low threshold for patient-provider contact at any time was emphasized. We used a multipronged approach: a non-stop telephone hotline was established whereby the transplant team can be contacted at any time, and more recently, several smartphone apps have been also made available to all KTRs (the IKEM Online app being the newest) (12). A team of trained nurses dedicated solely to the agenda of telehealth in outpatient KTRs managed the communication with patients and follow-ups in the prospective registry. All new cases and changes of health status were referred by the nurses to a specialized transplant nephrologist, who decided on the treatment, further management and frequency of further follow-up.

The aims of our proactive telehealth guided approach were the following:

1. To facilitate early COVID-19 diagnosis: to ensure high rates of early detection, KTRs were repeatedly instructed during outpatient visits, discharges from inpatient clinic, and through several mails, e-mail and text messaging campaigns to contact the center without hesitation in case of any suspicion for COVID-19 or if COVID-19 infection was confirmed.
2. To start treatment early: after the infection was confirmed, it was decided whether hospitalization was necessary or if outpatient management was possible, and treatment was initiated.
3. To monitor outpatient KTRs: outpatient KTRs were periodically checked-up remotely with the aims of identifying individuals with a progressive disease early on, ensuring a timely intensification of treatment.

Of note, the Czech Republic has a universal healthcare system based on a compulsory insurance model and telehealth was cost-free for KTRs, therefore limited financial resources should not be a major factor influencing the early/late referral rates.

### 2.2. Description of patient care at the transplantation center

The Institute for Clinical and Experimental Medicine (IKEM) is a high-volume transplant center that primarily covers geographic regions accounting for about 50% of the population of the Czech Republic (approximately 5 million). Patients transplanted at IKEM are followed-up regularly for the whole duration of their kidney transplant at our center. Since a substantial part of our KTRs live in more distant regions of the country, patient care is usually conducted in collaboration with a community nephrologist. However, the transplant center is consulted concerning most decisions and changes in KTR care ensuring continuity and homogeneous management.

### 2.3. Data collection

Anticipating a risk for significant morbidity and mortality at the onset of the pandemic, we established a new database recording each COVID-19 case including symptoms, immunosuppression regimen adjustments, infection management and patient outcomes.

The vast majority of COVID-19 infections were reported to our center before resolution of the active disease. However, to ensure complete data, the National Registry for Infectious Diseases (13) was searched to identify potentially missed cases. The National Registry for Infectious Diseases is a national government-run registry mandating the logging of all data regarding COVID-19 testing performed by all laboratories throughout the country. We discovered only 129 cases (13.5%) previously unregistered in our database and entered the data for these cases retrospectively. Mortality data were obtained from a government-run population registry.

Among the additional parameters gathered for the statistical analysis are KTRs’ age, sex, place of residence, date of transplantation, body mass index, immunosuppression use, all received COVID-19 vaccine doses, estimated glomerular filtration rate (eGFR), cause of end stage kidney disease, pretransplantation panel reactive antibodies, HLA mismatch, anti-HLA antibody positivity, and the history of various medical conditions that are needed for the calculation of Charlson comorbidity index (CCI). The Charlson comorbidity index is a weighted method of categorizing comorbidities where points are awarded for each known medical condition. A score of zero indicates no major comorbidities, while the higher the score, the higher the chance of adverse clinical events, mainly death (14).

### 2.4. Periods of the COVID-19 pandemic and the situation in the Czech Republic

**Figure 2:**
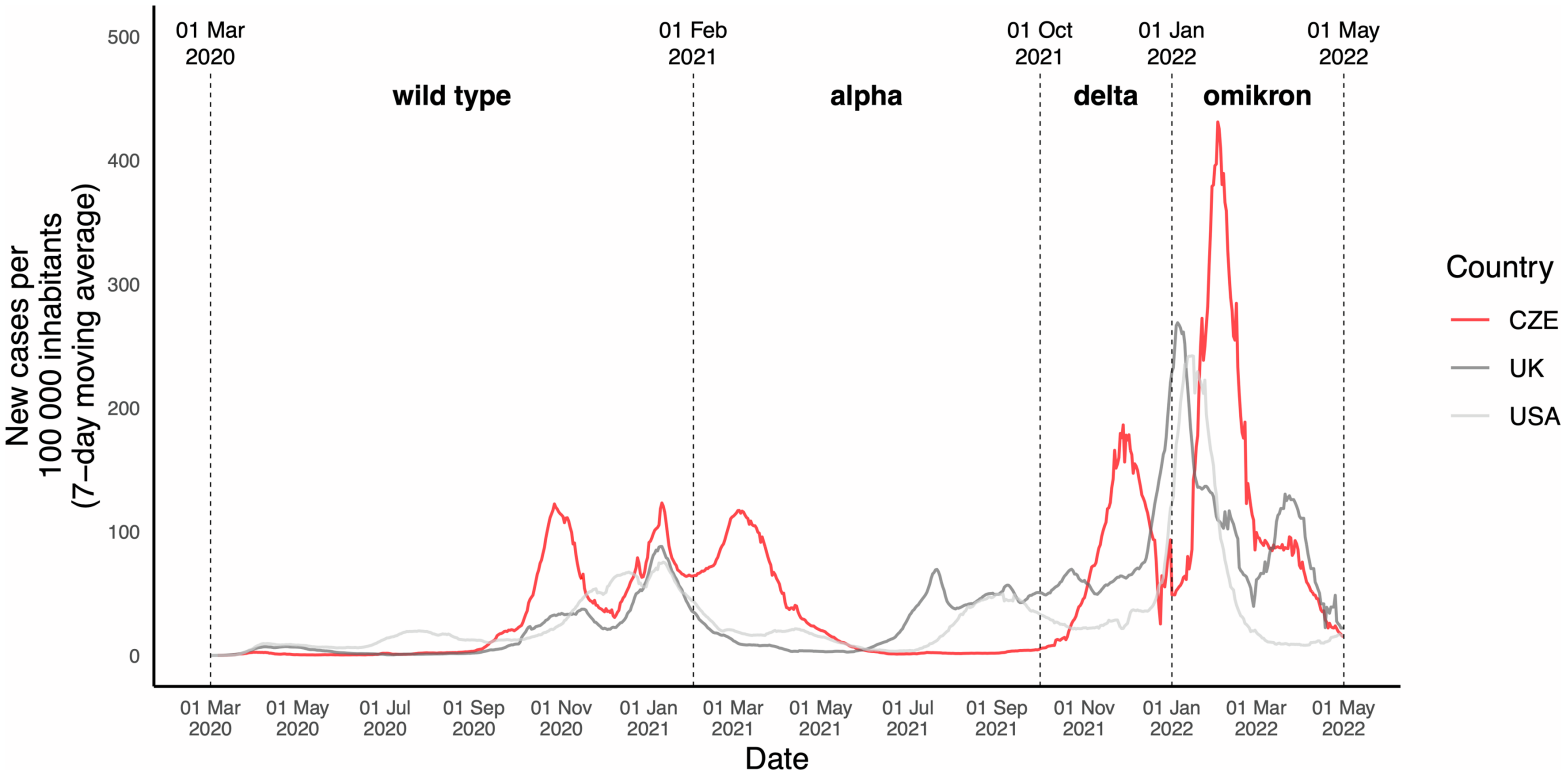
Overview of the dynamic of COVID-19 infection in the population during the study period. New cases of COVID-19 infection during the study period in the Czech Republic, the United Kingdom, and the United States. New cases are stated per 100,000 inhabitants. The periods are divided depending on the presumed dominant virus variant in the Czech Republic at the time. The UK and US data are shown solely to illustrate and draw a comparison and the dominant virus variants might have been different in the UK and US at each timepoint.

It has been shown that viral variants influenced the epidemiological and clinical characteristics of the disease (15, 16). To distinguish the individual pandemic periods and show effect of different viral strains, we divided the study period into 4 periods each dominated by a distinct viral strain. The periods are clearly distinguished by upheavals of the positive cases (Figure 2). The wild-type period ranged from March 1, 2020, to January 31, 2021; Alpha period ranged from February 1, 2021, to September 30, 2021; Delta period ranged from October 1, 2021, to December 31, 2021; while the Omicron period ranged from January 1, 2022, to the end of the study period (April 30, 2022). As viral RNA sequencing was performed in minority of cases, the division into individual periods were done solely by the dominant (expected) virus variant at the time of infection.

### 2.5. Early vs. late referral definition

**Figure 3:**
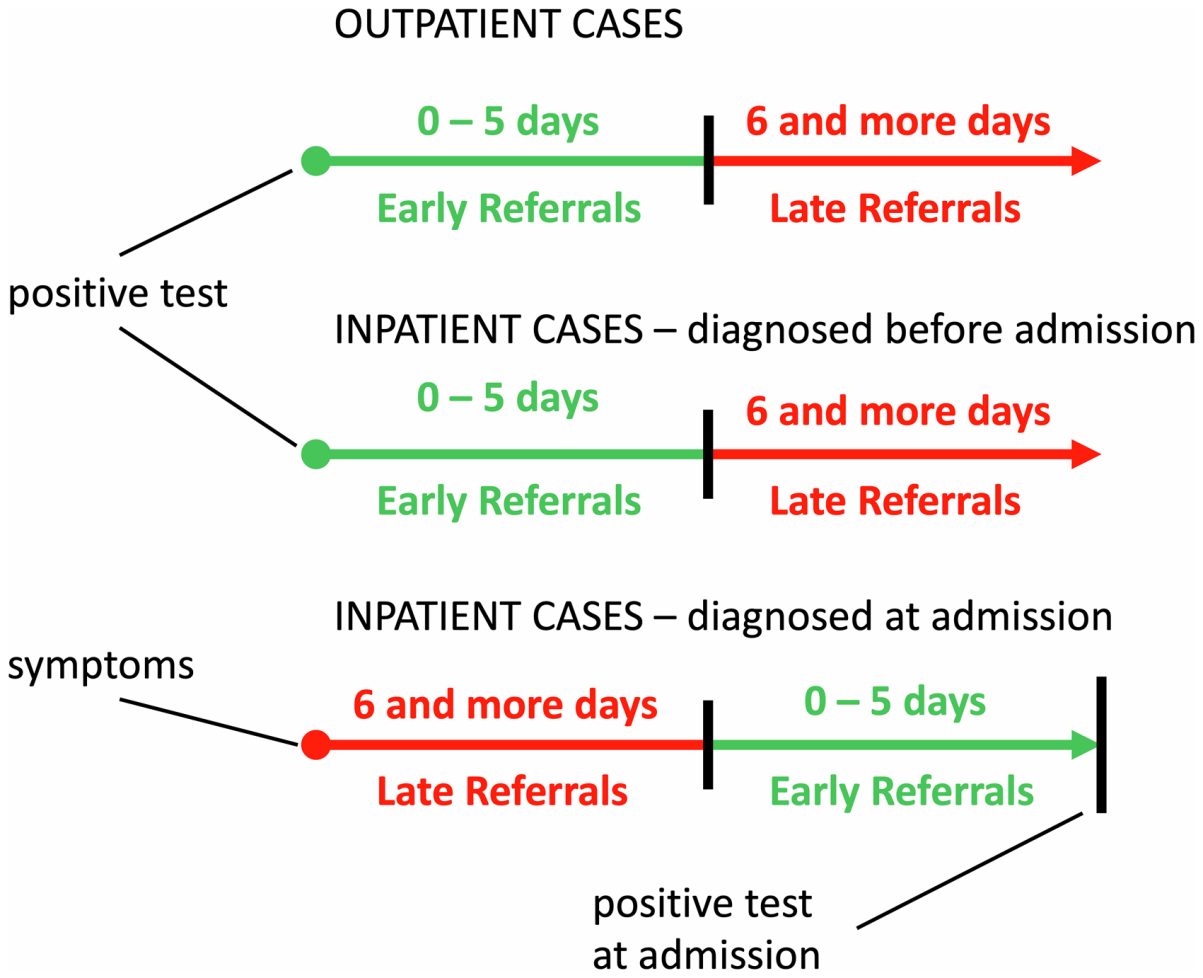
Definition of early and late referrals. Three different temporal scenarios for patients depending on whether they required hospitalization for COVID-19 (inpatient cases) or did not require hospitalization (outpatient cases) are depicted. Early and late referrals were defined in accordance with the COVID-19 biology. The first stage, characterized by viral infiltration and replication, usually lasts for 5–7 days. Afterwards, the pulmonary stage may begin in some patients. Because most interventions such as antiviral drugs are effective mostly within the first phase, we considered an early referral when a KTR contacted the transplant center within the period of the first stage of the disease. To reflect this, we defined early referral as a referral within the 5 days following a COVID-19 diagnosis. However, a diagnosis was made only at the time of hospital admission in a part of KTRs. We considered these KTRs being referred late except for KTRs in whom the need of hospitalization arose rapidly with less than 6 days from symptom onset to referral (i.e., they were therefore likely referred and treated while still in the first stage of the disease).

In order to assess the effect of early intervention, we defined the early and late referral in accordance with COVID-19 biology (17, 18). The first stage, characterized by viral infiltration and replication, usually lasts for 5–7 days. Afterwards, the pulmonary stage may develop. Because most interventions such as antiviral drugs are effective mostly within the first phase, we considered an early referral when a KTR contacted the transplant center within the period of the first stage of the disease. To reflect this, we defined early referral as a referral within the 5 days following a COVID-19 diagnosis. However, in a part of the KTRs, a diagnosis was made at the time of hospital admission. We considered these KTRs being referred late, with the exception of KTRs in whom the need of hospitalization arose rapidly with less than 6 days from symptom onset to referral (i.e., they were likely referred and treated while still in the first stage of the disease). In eight of these KTRs the onset of symptoms was unclear and thus could not be classified as early/late referrals and were excluded from the multivariable regression analysis. A graphical illustration of the temporal relations in the definition of early/late referral is depicted in Figure 3.

### 2.6. Statistical analysis

Statistics were calculated using R, version 4.1.1. Continuous variables are reported as medians with interquartile ranges (IQRs), categorical variables are reported as proportions. Wilcoxon rank sum test or Kruskal–Wallis test were used to compare continuous variables. Dunn’s post-hoc test was used to test intergroup differences. Chi-squared test or Fisher’s exact test were used to compare categorical variables. To test predictors of dichotomic variables, logistic regression was used. All tests were performed at the 5% level of significance.

## 3. Results

### 3.1. Demographics

The analysis, ranging from March 1, 2020, to April 30, 2022, included 912 KTRs with 955 confirmed COVID-19 infections representing all COVID-19 cases in KTRs followed-up at our transplant center during that period. Forty-three reinfections occurred in 42 KTRs. According to the definition of study periods based on the predominant circulating virus strain (Figure 2) there were 275, 199, 146, and 334 COVID-19 cases in wild-type, Alpha, Delta, and Omicron period, respectively.

An overview of characteristics is shown in Table 1 and a detailed overview is shown in Supplementary Table S1. Shorter time from transplantation, younger age, and lower CCI (19) of KTRs infected during the Omicron period are the most notable differences.

**Table 1:** Demographics and baseline characteristics.

Parameter	All infections (*n* = 954)	Wild-type period (*n* = 275)	Alpha period (*n* = 199)	Delta period (*n* = 146)	Omicron period (*n* = 334)	*p*-value
Male sex, *n* (%)	585 (61.3)	163 (59.3)	128 (64.3)	89 (61.0)	205 (61.4)	0.741
Age at COVID-19 diagnosis, median (IQR)	56.71 [47.47, 67.86]	57.85 [48.07, 68.60]	58.88 [47.97, 69.24]	58.63 [46.06, 68.22]	54.98 [46.74, 65.23]	0.054
Age category at COVID-19 diagnosis, *n* (%)						**0.027**
<50 years	310 (32.5)	86 (31.3)	61 (30.7)	47 (32.2)	116 (34.7)	
50–59 years	248 (26.0)	67 (24.4)	47 (23.6)	31 (21.2)	103 (30.8)	
60–69 years	220 (23.1)	62 (22.5)	47 (23.6)	38 (26.0)	73 (21.9)	
70–79 years	164 (17.2)	58 (21.1)	41 (20.6)	25 (17.1)	40 (12.0)	
>80 years	12 (1.3)	2 (0.7)	3 (1.5)	5 (3.4)	2 (0.6)	
Time between transplantation and COVID-19 diagnosis, median (IQR)	5.40 [2.13, 9.59]	6.03 [2.52, 11.22]	5.48 [2.67, 8.72]	5.60 [2.03, 10.69]	4.62 [1.81, 8.16]	**0.010** ^a^
Retransplantation, *n* (%)	124 (13.0)	37 (13.5)	20 (10.1)	19 (13.0)	48 (14.4)	0.545
Pretransplant PRA, median (IQR)	2.00 [0.00, 10.00]	2.00 [0.00, 16.00]	2.00 [0.00, 7.00]	2.00 [0.00, 11.00]	2.00 [0.00, 10.00]	0.320
HLA mismatch, median (IQR)	3.00 [2.00, 4.00]	3.00 [2.00, 4.00]	3.00 [2.00, 4.00]	3.00 [2.00, 4.00]	3.00 [2.00, 4.00]	0.727
Anti-HLA antibody positivity, *n* (%)	204 (34.8)	50 (35.5)	31 (26.1)	32 (36.0)	91 (38.4)	0.142
End stage kidney disease cause, *n* (%)						0.939
Polycystosis	167 (17.5)	47 (17.1)	34 (17.1)	28 (19.2)	58 (17.4)	
Glomerular disease	409 (42.9)	116 (42.2)	88 (44.2)	58 (39.7)	147 (44.0)	
Tubulointerstitial disease	115 (12.1)	37 (13.5)	18 (9.0)	17 (11.6)	43 (12.9)	
Diabetic kidney disease and/or vascular nephropathy	174 (18.2)	50 (18.2)	40 (20.1)	25 (17.1)	59 (17.7)	
Others	89 (9.3)	25 (9.1)	19 (9.5)	18 (12.3)	27 (8.1)	
Lives in close vicinity to transplantation center (in the capital city), *n* (%)	213 (22.3)	55 (20.0)	41 (20.6)	44 (30.1)	73 (21.9)	0.095

COVID-19, coronavirus disease 2019; IQR, interquartile range; PRA, panel-reactive antibodies; HLA, human leukocyte antigen.

a Dunn’s post-hoc test: wild-type vs. the Alpha variant (*p* = 0.304), wild-type vs. the Delta variant (*p* = 0.268), Alpha variant vs. Delta variant (*p* = 0.955), wild-type vs. Omicron variant (*p* = 0.005), Alpha variant vs. Omicron variant (*p* = 0.259), Delta variant vs. Omicron variant (*p* = 0.275). The bold values denote statistically significant results.

### 3.2. Patient outcomes

The COVID-19 severity as defined by the National Institutes of Health (NIH) (20) was generally mildest during the Omicron period. At least a moderate COVID-19 infection was found in 45% of the KTRs in the wild-type period, while only in 15.3% in the Omicron period. Given the challenges in clearly implicating COVID-19 as the primary cause of death, we decided to analyze 30 days (early) and 90 days (late) mortality after a COVID-19 diagnosis. The highest case fatality rate was observed during the Alpha period (12.6%) and it then decreased to 1.8% during the Omicron period. Patient outcomes during the individual periods are summarized in Table 2.

**Table 2:** Outcomes and treatment of COVID-19 infections.

Parameter	Whole cohort (*n* = 954)	Wild-type period (*n* = 275)	Alpha period (*n* = 199)	Delta period (*n* = 146)	Omicron period (*n* = 334)	*p*-value
COVID-19 severity (NIH)						**<0.001**
Asymptomatic infection, *n* (%)	65 (6.8)	16 (5.8)	15 (7.5)	10 (6.8)	24 (7.2)	
Mild illness, *n* (%)	584 (61.2)	135 (49.1)	90 (45.2)	100 (68.5)	259 (77.5)	
Moderate illness, *n* (%)	228 (23.9)	89 (32.4)	68 (34.2)	28 (19.2)	43 (12.9)	
Severe illness, *n* (%)	43 (4.5)	17 (6.2)	19 (9.5)	4 (2.7)	3 (0.9)	
Critical illness, *n* (%)	34 (3.6)	18 (6.5)	7 (3.5)	4 (2.7)	5 (1.5)	
Moderate or worse COVID-19 (NIH), *n* (%)	305 (32.0)	124 (45.1)	94 (47.2)	36 (24.7)	51 (15.3)	**<0.001**
Hospital admission						**<0.001**
Not admitted, *n* (%)	716 (75.1)	170 (61.8)	127 (63.8)	121 (82.9)	298 (89.2)	
Admitted—standard ward, *n* (%)	168 (17.6)	73 (26.5)	47 (23.6)	17 (11.6)	31 (9.3)	
Admitted—ICU, *n* (%)	70 (7.3)	32 (11.6)	25 (12.6)	8 (5.5)	5 (1.5)	
Death within 30 days from COVID-19 infection, *n* (%)	67 (7.0)	25 (9.1)	25 (12.6)	10 (6.8)	7 (2.1)	**<0.001**
Death within 90 days from COVID-19 infection, *n* (%)	81 (8.5)	30 (10.9)	28 (14.1)	13 (8.9)	10 (3.0)	**<0.001**
Maintenance immunosuppression reduced, *n* (%)	695 (72.8)	180 (65.5)	141 (70.9)	110 (75.3)	264 (79)	**0.002**
Whole immunosuppression stopped, *n* (%)	144 (15.1)	57 (20.7)	43 (21.6)	22 (15.1)	22 (6.6)	
Only MMF/MPA discontinuation, *n* (%)	525 (55)	116 (42.2)	90 (45.2)	83 (56.8)	236 (70.7)	
Others immunosuppression change, *n* (%)	26 (2.7)	7 (2.5)	8 (4)	5 (3.4)	6 (1.8)	
Targeted anti-COVID therapy, *n* (%)	482 (50.5)	20 (7.3)	66 (33.2)	132 (90.4)	264 (79)	**<0.001**
Molnupiravir, *n* (%)	159 (16.6)	0 (0)	0 (0)	0 (0)	159 (47.6)	**<0.001**
Remdesivir, *n* (%)	135 (14.1)	18 (6.5)	10 (5)	8 (5.5)	99 (29.6)	**<0.001**
^a^Monoclonal antibodies, *n* (%)	194 (20.3)	0 (0)	57 (28.6)	128 (87.7)	9 (2.7)	**<0.001**
Vaccinated with 2 doses prior to infection, *n* (%)	471 (49)	0 (0%)	46 (23%)	124 (85%)	301 (90%)	**<0.001**
Vaccinated with 3 doses prior to infection, *n* (%)	317 (33%)	0 (0%)	0 (0%)	55 (38%)	262 (78%)	**<0.001**

COVID-19, coronavirus disease 2019; NIH, National Institutes of Health; ICU, intensive care unit; MMF, mycophenolate mofetil; MPA, mycophenolic acid.

a The monoclonal antibodies used were bamlanivimab at first, which was later replaced with the combination of casivirimab and imdevimab. The bold values denote statistically significant results..

### 3.3. Predictors of death and severe COVID-19

The infected KTRs who survived more than 30 days following SARS-CoV-2 positivity were younger (56 vs. 71 years, *p* < 0.001), had shorter time from transplant to infection (5.3 vs. 7.4 years, *p* = 0.014), lower body mass index (28.1 vs. 30.4, *p* = 0.003) and presented with lower frequencies of various comorbidities and lower CCI (4 vs. 8, *p* < 0.001). Furthermore, survivors had a better allograft function (eGFR 48 vs. 31.8 mL/min/1.73m^2^, *p* < 0.001), more frequently contacted the transplant center early on after the diagnosis compared to non-survivors (78.1 vs. 49.3%, *p* < 0.001), and were more commonly vaccinated against COVID-19 compared to non-survivors (Table 3).

**Table 3:** Demographics of kidney transplant recipients with and without early (30 days) mortality.

Parameter	Did not die within 30D (*n* = 887)	Died within 30D (*n* = 67)	*p*-value
Male sex, *n* (%)	536 (60.4)	49 (73.1)	0.054
Age at COVID-19 diagnosis, median (IQR)	55.91 [46.51, 66.70]	70.74 [62.81, 73.74]	**<0.001**
Time from transplantation to COVID-19, median (IQR)	5.27 [2.07, 9.47]	7.44 [3.74, 11.34]	**0.014**
Retransplantation, *n* (%)	117 (13.2)	7 (10.4)	0.649
Pretransplantation PRA, median (IQR)	2.00 [0.00, 10.00]	2.00 [0.00, 9.50]	0.556
HLA mismatch, median (IQR)	3.00 [2.00, 4.00]	3.00 [2.00, 4.00]	0.125
Anti-HLA antibody positivity, *n* (%)	198 (35.6)	6 (20.0)	0.121
End stage kidney disease cause, *n* (%)			**0.001**
Polycystosis	157 (17.7)	10 (14.9)	
Glomerular disease	391 (44.1)	18 (26.9)	
Tubulointerstitial disease	107 (12.1)	8 (11.9)	
DKD and/or vascular nephropathy	149 (16.8)	25 (37.3)	
Others	83 (9.4)	6 (9.0)	
Lives in close vicinity to transplantation center (in the capital city), *n* (%)	204 (23.0)	9 (13.4)	0.097
Body mass index, median (IQR)	28.10 [24.92, 31.10]	30.40 [25.60, 34.10]	**0.003**
*Comorbidities, n (%)*			
Coronary artery disease, *n* (%)	125 (14.1)	22 (32.8)	**<0.001**
History of myocardial infarction, *n* (%)	42 (4.7)	10 (14.9)	**0.001**
History of congestive heart failure, *n* (%)	41 (4.6)	9 (13.4)	**0.005**
History of PVD, *n* (%)	41 (4.6)	17 (25.4)	**<0.001**
History of cerebrovascular disease, *n* (%)	53 (6.0)	5 (7.5)	0.821
Dementia, *n* (%)	4 (0.5)	5 (7.5)	**<0.001**
COPD, *n* (%)	37 (4.2)	6 (9.0)	0.130
Connective tissue disease, *n* (%)	46 (5.2)	2 (3.0)	0.614
Peptic ulcer, *n* (%)	60 (6.8)	15 (22.4)	**<0.001**
Liver disease, *n* (%)	27 (3.0)	3 (4.5)	0.775
History of malignity, *n* (%)	92 (10.4)	12 (17.9)	0.088
Diabetes mellitus, *n* (%)			**<0.001**
No diabetes	669 (75.4)	31 (46.3)	
Diabetes without complications	150 (16.9)	20 (29.9)	
Diabetes with complications	68 (7.7)	16 (23.9)	
Median Charlson comorbidity index (IQR)	4.00 [2.00, 6.00]	8.00 [6.00, 10.00]	**<0.001**
*Maintenance immunosuppression*			
Standard triple combination (tacrolimus, mycophenolate, corticosteroids)	396 (44.6)	12 (17.9)	**<0.001**
Any triple combination	443 (49.9)	14 (20.9)	**<0.001**
Last known eGFR before COVID-19 (mL/min/1.73m^2^), median (IQR)	48 [34.2, 62.4]	31.8 [21, 45]	**<0.001**
Late or no referral to transplant center, *n* (%)^a^	194 (21.9)	34 (50.7)	**<0.001**
Vaccination			**0.001**
Unvaccinated	437 (49.3)	46 (68.7)	
Vaccinated with 2 doses while infected	142 (16.0)	12 (17.9)	
Vaccinated with 3 doses while infected	308 (34.7)	9 (13.4)	

COPD, chronic obstructive pulmonary disease; COVID-19, coronavirus disease 2019; D, day; DKD, diabetic kidney disease; IQR, interquartile range; PRA, panel-reactive antibodies; PVD, peripheral vascular disease; eGFR, estimated glomerular filtration rate.

a There are 4 and 4 missing values in both columns (0.45% and 5.9% respectively). The bold values denote statistically significant results.

A logistic regression model of risk factors of death within 30 days was constructed (univariable associations are shown in Supplementary Table S2, multivariable model is shown in Table 4). The multivariable model showed the following independent risk factors of 30 day mortality: age at COVID-19 infection (OR 1.09, 95% CI 1.05–1.13, *p* < 0.001), body mass index (OR 1.06, 95% CI 1.01–1.12, *p* = 0.03), graft function (eGFR at the latest check-up prior to COVID-19 infection in mL/min/1.73m^2^, OR 0.96, 95% CI 0.94–0.98, *p* < 0.001), and late referral to the transplant center (OR 2.08, 95% CI 1.08–3.98, *p* = 0.027).

**Table 4:** Multivariable logistic regression for 30 days mortality.

Predictor	OR	95% CI	*p*-value
Male sex	1.78	0.94, 3.51	0.085
Age at COVID-19 diagnosis	1.09	1.05, 1.13	**<0.001**
Body mass index	1.06	1.01, 1.12	**0.030**
*Diabetes*			
No diabetes	ref.	ref.	ref.
Diabetes without complications	1.20	0.58, 2.41	0.6
Diabetes with complications	1.60	0.69, 3.57	0.3
Standard triple therapy (TAC + MMF/MPA + CS)	0.75	0.35, 1.52	0.4
Last known eGFR before COVID-19	0.96	0.94, 0.98	**<0.001**
*Virus variant period*			
Wild-type period	ref.	ref.	ref.
Alpha period	1.81	0.89, 3.73	0.1
Delta period	0.76	0.28, 1.91	0.6
Omicron period	0.40	0.14, 1.00	0.06
Late referral	2.08	1.08, 3.98	**0.027**

OR, odds ratio; 95% CI, 95% confidence interval; COVID-19, coronavirus disease 2019; TAC, tacrolimus; MMF, mycophenolate mofetil; MPA, mycophenolic acid; CS, corticosteroids; eGFR, estimated glomerular filtration rate; ref., reference. The bold values denote statistically significant results.

Furthermore, clinical characteristics and univariable logistic regression associations of those who died within 90 days, were admitted to ICU, or developed at least moderate a COVID-19 disease, are presented in Supplementary Tables S3, S4.

### 3.4. Role of the early referral to the transplant center

To assess the performance of our telemedicine guided approach towards early diagnostics and management of COVID-19 cases, we evaluated the association between early referral to the transplant center and mortality. We found that KTRs who were referred early had a lower risk of death within 30 days (4.5% vs. 13.6%, *p* < 0.001) and 90 days (5.6% vs. 16.4%, *p* < 0.001) following COVID-19 diagnosis.

Clinical characteristics of early and late referrals are presented in Table 5. Notably, KTRs who contacted the transplant center late were older (59.4 vs. 55.9 years, *p* = 0.007), had longer time after transplantation (7.2 vs. 4.7 years, *p* < 0.001), lived farther from the transplant center (85.9 vs. 74.9%, *p* < 0.001), and were diabetic (32.7 vs. 24.5%, *p* = 0.011). Accordingly, they presented a higher CCI (5 vs. 4, *p* = 0.009).

**Table 5:** Demographics, baseline characteristics and treatment of early and late referrals.

Parameter	Late referral (*n* = 228)	Early referral (*n* = 726)	*p*-value
Male recipient, *n* (%)	136 (61.8)	444 (61.2)	0.922
Median age at COVID-19, years (IQR)	59.35 [49.62, 69.60]	55.89 [46.55, 67.02]	**0.007**
Median time from transplantation to COVID-19, years (IQR)	7.15 [3.70, 12.09]	4.72 [1.95, 8.78]	**<0.001**
Lives in close vicinity to transplantation center, *n* (%)	31 (14.1)	182 (25.1)	**0.001**
Median body mass index, kg/m^2^ (IQR)	29.00 [25.10, 31.60]	28.00 [24.90, 31.30]	0.128
*Comorbidities*			
Diabetes mellitus, *n* (%)			**0.011**
No diabetes	148 (67.3)	548 (75.5)	
Diabetes without complications	54 (24.5)	114 (15.7)	
Diabetes with complications	18 (8.2)	64 (8.8)	
Median Charlson comorbidity index (IQR)	5.00 [3.00, 7.00]	4.00 [2.00, 6.00]	**0.009**
Standard triple immunosuppression combination (tacrolimus, MMF/MPA, corticosteroids), *n* (%)	73 (33.2)	335 (46.1)	**0.001**
Vaccination status			**<0.001**
Unvaccinated	178 (78)	305 (42)	
Vaccinated with 2 doses prior to infection	24 (11)	130 (18)	
Vaccinated with 3 doses prior to infection	291 (40)	26 (11)	
Vaccination status (wild-type period excluded)^a^			**<0.001**
Unvaccinated	59 (55.7)	148 (26)	
Vaccinated with 2 doses prior to infection	22 (20.8)	130 (22.8)	
Vaccination with 3 doses prior to infection	25 (23.6)	291 (51.1)	
Virus variant period, *n* (%)			**<0.001**
Wild-type period	114 (51.8)	157 (21.6)	
Alpha period	60 (27.3)	137 (18.9)	
Delta period	16 (7.3)	128 (17.6)	
Omicron period	30 (13.6)	304 (41.9)	
Targeted anti-COVID-19 treatment, *n* (%)	39 (17.1)	443 (61)	**<0.001**
Molnupiravir	8 (3.5)	151 (20.8)	**0.002**
Remdesivir	21 (9.2)	114 (15.7)	**0.004**
Monoclonal antibodies	10 (4.4)	184 (25.3)	**<0.001**
Withdrawal of all immunosuppression	58 (25.4)	86 (11.8)	**<0.001**
Isolated mycophenolate withdrawal	44 (19.3)	481 (66.3)	**<0.001**

COVID-19, coronavirus disease 2019; IQR, interquartile range; MMF, mycophenolate mofetil; MPA, mycophenolic acid.

a Cases of COVID-19 infection during the wild-type period (*n* = 275) are excluded in this calculation of proportions of vaccinated patients as no patient could be vaccinated during the wild-type period. The bold values denote statistically significant results.

As shown above, after multivariable adjustment for confounders, late referral to the transplant center was independently associated with death within 30 days following a COVID-19 diagnosis (OR 2.08, 95% CI 1.08–3.96, *p* = 0.027; Table 4).

Additional sensitivity analyses further support the association between early referral to the transplant center and lower mortality (Supplementary Table S5). To explore this link, we constructed a multivariable model that included vaccination status. Vaccination status was previously not included in the multivariable model due to its high degree of collinearity with the period variable. However, since vaccination affects mortality and late referrals were less likely to be vaccinated with 2 doses (10% vs. 17.9%) and with 3 doses (11.4% vs. 40.1%) at the time of infection, it was necessary to show a model adjusted for vaccination status despite the collinearity. Next, another complementary model was made to better address the impact of the Omicron period specifically. We decided for this analysis because: (1) the Omicron variant is biologically and clinically distinct, (2) the clinical characteristics in our cohort were similar in the first three periods compared to Omicron, (3) the most infection cases were registered during the Omicron period while having the lowest death rate, and (4) the early referral rate was highest during the Omicron period. Finally, a model with the same variable selection as the main model but with the exclusion of KTRs infected during the wild-type period was calculated, as in general neither vaccination nor specific anti-COVID treatment was available in that period.

Altogether, late referral to the transplant center was an independent risk factor of death within 30 days following a COVID-19 diagnosis in all four presented multivariable models.

### 3.5. Therapeutic interventions and vaccination during individual periods

The development of therapeutic interventions during individual pandemic periods is summarized in Table 2.

The rate of immunosuppression reduction generally increased during the pandemic (65.5% in the wild-type period to 79.3% in the Omicron period). However, the need for a complete withdrawal decreased from 20.7% during the wild-type period to 6.6% during the Omicron period. Short-term isolated reduction/withdrawal of mycophenolate was the most common immunosuppression adjustment and increased from 42.2% during the wild-type period to 70.7% during the Omicron period.

As new antivirals were being developed, the availability and indication criteria changed during the individual periods of the pandemic and so did their use. Concerning vaccination, 23% of KTRs were fully vaccinated (i.e., received two doses) prior to being infected during the Alpha period, while in the Delta period 85% were fully vaccinated and 38% received a booster dose prior to infection. During the Omicron period 90% of KTRs were fully vaccinated and 78% received a booster dose prior to being infected.

Most of the targeted COVID-19 treatment options were directly dependent on early referrals to the transplant center. This is demonstrated by a much higher proportion of KTRs overall receiving targeted COVID-19 treatment (61% vs. 17.1%; *p* < 0.001), as well as individual treatments such as remdesivir, molnupiravir and monoclonal antibodies (Table 5).

## 4. Discussion

In this single-center observational cohort study analyzing prospectively gathered data, we describe the course of the COVID-19 pandemic on a large cohort of vulnerable KTRs. We show that COVID-19 positive patients who were referred to the transplant center and therefore were managed earlier and were more closely monitored, had a lower risk of death from COVID-19. To the best of our knowledge, this is the largest report on the effect of telehealth on a hard endpoint, such as COVID-related mortality, in a high-risk population.

This benefit of the early referral might be the result of a combination of several mechanisms, namely the timely administration of targeted treatment. As even the most efficacious antivirals are significantly less effective when they cannot reach the patients within an appropriate time period, we believe that early referral must be viewed as an important gatekeeping mechanism which enables further treatment. This is also demonstrated by the lower rate of application of targeted antiviral drugs in the late referral group. The first stage of COVID-19 is characterized by viral infiltration and replication that usually lasts for 5–7 days, and it is presumed that antiviral treatment has the largest impact within this time frame (17, 18). Therefore, it is generally recommended to initiate the specific treatment with antivirals and monoclonal antibodies as soon as possible (20). Furthermore, it is likely that early identification and initiation of therapy in critically ill patients with COVID-19 increases their chances of survival. For example, it has been reported that early oxygen therapy is associated with better outcomes in critically ill COVID-19 patients (21–23). Additionally, specifically for KTRs, a timely adjustment of maintenance immunosuppression may facilitate virus elimination (24). Therefore, it has been suggested that an early reduction of immunosuppression could be beneficial. What would be the ideal strategy, however, remains unclear (25).

We believe that the aforementioned factors are some of the reasons behind the mortality reduction in the COVID-19 infected KTRs who contacted the transplant center early and the use of telehealth has thus shown to be a useful tool for providing patient care during the pandemic. It is, however, important to select an appropriate tailored modality for remote patient management. Despite a recent explosion of smart technologies, a simple telephone call might yield better results than modern smartphone apps, particularly in some patient subpopulations, e.g., the elderly (26).

Based on our findings, we propose several measures that should be taken to reduce the impacts of an infectious disease outbreak in vulnerable patient populations. First, it is necessary to give clear instructions to the patients when they should contact their healthcare provider and to facilitate this process by ensuring a low threshold for patient-provider communication. This applies especially for subgroups that are least likely to contact their healthcare providers, such as older patients and patients living farther from the center. Furthermore, collaboration with community healthcare providers and specialists is key for specific vulnerable patient populations such as KTRs, where a transplant specialist should be consulted when important decisions need to be made about the therapy of an immunosuppressed KTR (20). Finally, the use of telehealth should be considered for remote monitoring, management adjustments, timely recognition of potential complications, and adoption of other appropriate measures.

This report does not aim to evaluate vaccine effectiveness as biases such as fluctuation of the background risk of infection and virus evolution must be carefully assessed to limit incorrect conclusions (27). With the rapid evolution of viral biology, treatment options and vaccination strategies, the assessment of vaccine effectiveness becomes ever more complicated. However, it has been previously reported by us and others that vaccines are effective in the reduction of infection rates in KTRs, although the effectiveness is lower than in the general population (28, 29).

Among the strengths of our study are the use of reliable epidemiologic data from the central nationwide registry with mandatory reporting (13), and the large cohort in a high-volume transplant center. The single-center aspect of the study is advantageous, as it inherently results in a rather homogenous approach towards pre-hospitalization patient management based on internal guidelines.

Among the limitations of this study is its retrospective design, which renders it impossible to analyze the efficacy of different treatment options and we intentionally avoid making such evaluations. Furthermore, viral sequencing was done only in a minority of cases, therefore the division into individual pandemic periods was done solely based on the presumed dominant virus variant at the time of infection. Also, although it can be presumed that socio-economic status impacts both COVID-19 related mortality and the frequency of early contact with the transplant center, this could not be assessed as the required data were not available.

Lastly, clearly multiple confounders impact COVID-19 mortality, including comorbidities, behavioral characteristics, as well as the evolution of disease and treatment in time. As the pandemic developed, patient outcomes improved and at the same time the rates of early referral increased. However, the standard epidemiological approach used should allow to adjust for these confounders, including the effect of time (27, 28).

## 5. Conclusion

In conclusion, we found an association between an early referral, timely intervention, and monitoring of kidney transplant recipients via telehealth and a decreased risk of death. Implementing policies aimed at the promotion and facilitation of communication between kidney transplant recipients and their transplant specialists, early identification of infection cases and remote monitoring may be promising tools in the reduction of serious patient outcomes during future infectious disease outbreaks. Furthermore, these results can be likely extrapolated to other vulnerable populations such as patients with cancer, diabetes or autoimmune diseases requiring the use of immunosuppression.

## Data availability statement

The raw data supporting the conclusions of this article will be made available by the authors, without undue reservation.

## Ethics statement

Ethical approval was not required for the studies involving humans because Institutional review board/Ethics committee approval is not required for anonymous retrospective observational studies under the current legislature in the Czech Republic. The studies were conducted in accordance with the local legislation and institutional requirements. Written informed consent for participation was not required from the participants or the participants’ legal guardians/next of kin in accordance with the national legislation and institutional requirements because Written informed consent is not required for anonymous retrospective observational registry-based studies.

## Author contributions

IZ, VP, KJ, IM, FH, and OV were responsible for the conceptualization of the study, participated in the acquisition of resources, and contributed to the review and editing of the final article. IZ, VP, KJ, IM, and FH took part in data curation. IZ, VP, and IM were responsible for formal analysis and validated the results. OV was responsible for funding acquisition, project administration, and supervision. IZ, VP, and OV were responsible for the methodology and wrote the original draft. IM and FH were responsible for the software support. VP and IM had taken part in the visualization. IZ and VP contributed equally to this work. All authors contributed to the article and approved the submitted version.

## Funding

This research was supported by the Ministry of Health of the Czech Republic NU22-C-126 and the project National Institute for Research of Metabolic and Cardiovascular Diseases (Programme EXCELES, Project No. LX22NPO5104)—funded by the European Union—Next Generation EU.

## Conflict of interest

The authors declare that the research was conducted in the absence of any commercial or financial relationships that could be construed as a potential conflict of interest.

## Publisher’s note

All claims expressed in this article are solely those of the authors and do not necessarily represent those of their affiliated organizations, or those of the publisher, the editors and the reviewers. Any product that may be evaluated in this article, or claim that may be made by its manufacturer, is not guaranteed or endorsed by the publisher.

## Abbreviations

BMI: body mass index
CCI: Charlson comorbidity index
Covid-19: coronavirus disease 2019
eGFR: estimated glomerular filtration rate
ICU: intensive care unit
IQR: interquartile range
NIH: National Institutes of Health
KTR: kidney transplant recipient
SARS-CoV-2: severe acute respiratory syndrome coronavirus 2
