# Supplementary material for: Early referring saved lives in kidney transplant recipients with COVID-19: a beneficial role of telemedicine
Source: Front Med (Lausanne). 2023 Sep 19;10:1252822. doi: 10.3389/fmed.2023.1252822 (PMC10546052; doi:10.3389/fmed.2023.1252822)
Supplement: Supplementary file 1 [file Table_1.DOCX]

**Supplementary Table 1. Comorbidities and maintenance immunosuppression.**

| **Parameter** | **Whole cohort**  **(n = 954)** | **Wild-type period**  **(n = 275)** | **Alpha period**  **(n = 199)** | **Delta period**  **(n = 146)** | **Omicron period**  **(n = 334)** | **p-value** |
| --- | --- | --- | --- | --- | --- | --- |
| **Median body mass index, kg/m2 (IQR)** | 28.20  [25.00, 31.40] | 28.75  [25.52, 32.00] | 27.80  [24.90, 31.60] | 28.40  [25.30, 30.90] | 27.80  [24.63, 30.90] | 0.140 |
| **Comorbidities** |  |  |  |  |  |  |
| **Coronary artery disease, n (%)** | 147 (15.4) | 36 (13.1) | 31 (15.6) | 27 (18.5) | 53 (15.9) | 0.521 |
| **History of myocardial infarction, n (%)** | 52 (5.5) | 16 (5.8) | 12 (6.0) | 8 (5.5) | 16 (4.8) | 0.922 |
| **History of congestive heart failure, n (%)** | 50 (5.2) | 18 (6.5) | 11 (5.5) | 9 (6.2) | 12 (3.6) | 0.384 |
| **History of peripheral vascular disease, n (%)** | 58 (6.1) | 17 (6.2) | 19 (9.5) | 10 (6.8) | 12 (3.6) | **0.047** |
| **History of cerebrovascular disease, n (%)** | 58 (6.1) | 16 (5.8) | 11 (5.5) | 11 (7.5) | 20 (6.0) | 0.877 |
| **Dementia, n (%)** | 9 (0.9) | 4 (1.5) | 2 (1.0) | 0 (0.0) | 3 (0.9) | 0.537 |
| **Chronic obstructive pulmonary disease, n (%)** | 43 (4.5) | 14 (5.1) | 14 (7.0) | 4 (2.7) | 11 (3.3) | 0.146 |
| **Connective tissue disease, n (%)** | 48 (5.0) | 14 (5.1) | 10 (5.0) | 11 (7.5) | 13 (3.9) | 0.420 |
| **Peptic ulcer, n (%)** | 75 (7.9) | 18 (6.5) | 20 (10.1) | 9 (6.2) | 28 (8.4) | 0.444 |
| **Liver disease, n (%)** | 30 (3.1) | 11 (4.0) | 8 (4.0) | 4 (2.7) | 7 (2.1) | 0.485 |
| **Diabetes mellitus, n (%)** |  |  |  |  |  | 0.171 |
| - **no diabetes** | 700 (73.4) | 197 (71.6) | 137 (68.8) | 103 (70.5) | 263 (78.7) |  |
| - **diabetes without complications** | 170 (17.8) | 50 (18.2) | 41 (20.6) | 28 (19.2) | 51 (15.3) |  |
| - **diabetes with complications** | 84 (8.8) | 28 (10.2) | 21 (10.6) | 15 (10.3) | 20 (6.0) |  |
| **Cancer, n (%)** | 104 (10.9) | 40 (14.5) | 15 (7.5) | 21 (14.4) | 28 (8.4) | **0.018** |
| **Median Charlson Comorbidity Index (IQR)** | 4.00 [3.00, 6.00] | 4.00 [3.00, 7.00] | 4.00 [3.00, 7.00] | 4.00 [3.00, 7.00] | 4.00 [2.00, 6.00] | **0.010*** |
| **Donor type** |  |  |  |  |  | 0.456 |
| **Donation after brain death, standard criteria donor, n (%)** | 292 (33.0) | 88 (35.9) | 53 (28.8) | 41 (29.5) | 110 (34.8) |  |
| **Extended criteria donor or donation after circulatory death, n (%)** | 382 (43.2) | 94 (38.4) | 88 (47.8) | 64 (46.0) | 136 (43.0) |  |
| **Living donation, n (%)** | 210 (23.8) | 63 (25.7) | 43 (23.4) | 34 (24.5) | 70 (22.2) |  |
| **Maintenance immunosuppression** |  |  |  |  |  |  |
| **Standard triple combination (tacrolimus, MMF/MPA, corticosteroids)** | 408 (42.8) | 105 (38.2) | 77 (38.7) | 54 (37.0) | 172 (51.5) | **0.001** |
| **Any triple combination** | 457 (47.9) | 125 (45.5) | 84 (42.2) | 64 (43.8) | 184 (55.1) | **0.011** |
| **Tacrolimus** | 566 (59.3) | 159 (57.8) | 105 (52.8) | 86 (58.9) | 216 (64.7) | 0.051 |
| **Ciclosporin A** | 50 (5.2) | 18 (6.5) | 12 (6.0) | 10 (6.8) | 10 (3.0) | 0.148 |
| **MMF/MPA** | 509 (53.4) | 149 (54.2) | 90 (45.2) | 70 (47.9) | 200 (59.9) | **0.005** |
| **Corticosteroids** | 580 (60.8) | 162 (58.9) | 109 (54.8) | 93 (63.7) | 216 (64.7) | 0.109 |
| **mTOR inhibitor** | 30 (3.1) | 11 (4.0) | 3 (1.5) | 5 (3.4) | 11 (3.3) | 0.480 |
| **Belatacept** | 6 (0.6) | 1 (0.4) | 1 (0.5) | 2 (1.4) | 2 (0.6) | 0.649 |

**Abbreviations:** IQR, interquartile range; MMF, mycophenolate mofetil; MPA, mycophenolic acid; mTOR, mechanistic target of rapamycin.

* Dunn’s post-hoc test: Wild-type vs. Alpha variant (p = 0.981), Wild-type vs. Delta variant (p = 1), Alpha variant vs. Delta variant (p = 1), Wild-type vs. Omicron variant (p = 0.035), Alpha Variant vs. Omicron variant (p = 0.039), Delta variant vs. Omicron variant (p = 0.027)

**Supplementary Table 2. Univariable predictors of early (30 days) mortality**

| **Predictor** | **OR** | **95% CI** | **p-value** |
| --- | --- | --- | --- |
| **Male sex** | 1.78 | 1.04, 3.19 | **0.042** |
| **Age at COVID-19** | 1.11 | 1.08, 1.14 | **<0.001** |
| **Age at COVID-19 (categories)** |  |  |  |
| **< 50 years** | — | — |  |
| **50 – 60 years** | 5.80 | 1.48, 38.3 | **0.025** |
| **60 – 70 years** | 15.4 | 4.43, 97.1 | **<0.001** |
| **70 – 80 years** | 35.9 | 10.7, 224 | **<0.001** |
| **> 80 years** | 110 | 20.2, 871 | **<0.001** |
| **Years from transplantation** | 1.03 | 0.99, 1.07 | 0.12 |
| **End stage kidney disease cause** |  |  |  |
| **Polycystosis** | — | — |  |
| **Glomerular disease** | 0.72 | 0.33, 1.66 | 0.4 |
| **Tubulointerstitial disease** | 1.17 | 0.44, 3.07 | 0.7 |
| **Diabetic kidney disease and/or vascular nephropathy** | 2.63 | 1.26, 5.93 | **0.013** |
| **Others** | 1.13 | 0.37, 3.17 | 0.8 |
| **Lives in close vicinity to transplantation center** | 0.52 | 0.24, 1.02 | 0.074 |
| **Body mass index** | 1.07 | 1.03, 1.12 | **0.001** |
| **Coronary artery disease** | 2.98 | 1.70, 5.08 | **<0.001** |
| **History of myocardial infarction** | 3.53 | 1.60, 7.15 | **<0.001** |
| **History of congestive heart failure** | 3.20 | 1.40, 6.64 | **0.003** |
| **History of peripheral vascular disease** | 7.02 | 3.65, 13.1 | **<0.001** |
| **History of cerebrovascular disease** | 1.27 | 0.43, 3.01 | 0.6 |
| **Dementia** | 17.8 | 4.60, 73.5 | **<0.001** |
| **Chronic obstructive pulmonary disease** | 2.26 | 0.83, 5.20 | 0.076 |
| **Peptic ulcer disease** | 3.98 | 2.06, 7.33 | **<0.001** |
| **Liver disease** | 1.49 | 0.35, 4.38 | 0.5 |
| **Diabetes** |  |  |  |
| - **no diabetes** | — | — |  |
| - **diabetes without complications** | 2.88 | 1.57, 5.15 | **<0.001** |
| - **diabetes with complications** | 5.08 | 2.59, 9.64 | **<0.001** |
| **History of cancer** | 1.89 | 0.93, 3.54 | 0.060 |
| **Charlson Comorbidity Index** | 1.41 | 1.30, 1.53 | **<0.001** |
| **Standard triple therapy (TAC + MMF/MPA + CS)** | 0.27 | 0.14, 0.49 | **<0.001** |
| **Last known eGFR** | 0.96 | 0.94, 0.97 | <0.001 |
| **Virus periods** | — | — |  |
| **Wild-type period** | — | — |  |
| **Alpha period** | 1.44 | 0.80, 2.59 | 0.2 |
| **Delta period** | 0.74 | 0.33, 1.53 | 0.4 |
| **Omicron period** | 0.21 | 0.08, 0.48 | **<0.001** |
| **Wild-type + Alpha + Delta vs. Omicron** | 0.20 | 0.08, 0.41 | **<0.001** |
| **Late contact*** | 3.32 | 1.96, 5.58 | **<0.001** |
| **Vaccination** | 0.44 | 0.26, 0.75 | **0.003** |
| - **unvaccinated** | — | — |  |
| - **vaccinated with 2 doses while infected** | 0.80 | 0.40, 1.51 | 0.5 |
| - **vaccinated with 3 doses while infected** | 0.28 | 0.13, 0.55 | **<0.001** |

**Abbreviations:** OR, odds ratio; 95% CI, 95% confidence interval; COVID-19, coronavirus disease 2019, TAC, tacrolimus; MMF, mycophenolate mofetil; MPA, mycophenolic acid; CS, corticosteroids.

**Note:** 8 missing values.

**Supplementary Table 3. Demographics and baseline characteristics – late (90 days) mortality, intensive care unit admission, and at least moderate COVID-19 severity.**

| **Parameter** | **Death within 90 days** | | | **Admission to intensive care unit** | | | **At least moderate severity of COVID-19** | | |
| --- | --- | --- | --- | --- | --- | --- | --- | --- | --- |
|  | No (n = 873) | Yes (n = 81) | p-value | No (n = 884) | Yes (n = 70) | p-value | No (n = 649) | Yes (n = 305) | p-value |
| **Male recipient, n (%)** | 524 (60.0) | 61 (75.3) | **0.010** | 537 (60.7) | 48 (68.6) | 0.243 | 380 (58.6) | 205 (67.2) | **0.013** |
| **Median age at COVID-19, years (IQR)** | 55.72 [46.37, 66.36] | 70.74 [63.74, 74.31] | **<0.001** | 56.06 [46.54, 67.04] | 66.19 [58.08, 72.37] | **<0.001** | 53.35 [45.06, 63.97] | 64.05 [55.72, 71.20] | **<0.001** |
| **Median time from transplantation to COVID-19, years (IQR)** | 5.26 [2.06, 9.44] | 7.44 [3.68, 11.94] | **0.008** | 5.36 [2.13, 9.49] | 5.77 [2.09, 12.83] | 0.466 | 4.74 [1.95, 8.84] | 6.88 [2.98, 11.20] | **<0.001** |
| **Retransplantation, n (%)** | 117 (13.4) | 7 (8.6) | 0.296 | 118 (13.3) | 6 (8.6) | 0.337 | 88 (13.6) | 36 (11.8) | 0.516 |
| **Median pretransplantation PRA (IQR)** | 2.00 [0.00, 12.00] | 2.00 [0.00, 8.00] | 0.476 | 2.00 [0.00, 11.00] | 2.00 [0.00, 8.50] | 0.605 | 2.00 [0.00, 10.00] | 2.00 [0.00, 20.00] | 0.050 |
| **Median HLA mismatch (IQR)** | 3.00 [2.00, 4.00] | 3.00 [2.50, 4.00] | 0.461 | 3.00 [2.00, 4.00] | 3.00 [3.00, 4.00] | 0.594 | 3.00 [2.00, 4.00] | 3.00 [2.00, 4.00] | 0.541 |
| **Anti-HLA antibodies positive, n (%)** | 196 (35.7) | 8 (21.6) | 0.118 | 197 (35.9) | 7 (18.9) | 0.055 | 157 (36.0) | 47 (31.3) | 0.348 |
| **End stage kidney disease cause, n (%)** |  |  | **<0.001** |  |  | 0.113 |  |  | **<0.001** |
| - **Polycystosis** | 154 (17.6) | 13 (16.0) |  | 155 (17.5) | 12 (17.1) |  | 113 (17.4) | 54 (17.7) |  |
| - **Glomerular disease** | 388 (44.4) | 21 (25.9) |  | 383 (43.3) | 26 (37.1) |  | 298 (45.9) | 111 (36.4) |  |
| - **Tubulointerstitial disease** | 106 (12.1) | 9 (11.1) |  | 109 (12.3) | 6 (8.6) |  | 79 (12.2) | 36 (11.8) |  |
| - **Diabetic kidney disease and/or vascular nephropathy** | 143 (16.4) | 31 (38.3) |  | 153 (17.3) | 21 (30.0) |  | 93 (14.3) | 81 (26.6) |  |
| - **Others** | 82 (9.4) | 7 (8.6) |  | 84 (9.5) | 5 (7.1) |  | 66 (10.2) | 23 (7.5) |  |
| **Late or no contact to transplant center, n (%)*** | 188(21.5) | 40 (49.4) | **<0.001** | 193 (21.8) | 35 (50.0) | **<0.001** | 115 (17.7) | 113 (37.0) | **<0.001** |
| **Median body mass index, kg/m2 (IQR)** | 28.10 [24.90, 31.10] | 30.40 [25.80, 35.00] | **<0.001** | 28.10 [24.90, 31.10] | 30.55 [26.85, 34.05] | **<0.001** | 27.80 [24.70, 30.80] | 29.30 [25.48, 32.60] | **<0.001** |
| **Comorbidities, n (%)** |  |  |  |  |  |  |  |  |  |
| **Coronary artery disease, n (%)** | 120 (13.7) | 27 (33.3) | **<0.001** | 127 (14.4) | 20 (28.6) | **0.003** | 79 (12.2) | 68 (22.3) | **<0.001** |
| **History of myocardial infarction, n (%)** | 37 (4.2) | 15 (18.5) | **<0.001** | 45 (5.1) | 7 (10.0) | 0.142 | 23 (3.5) | 29 (9.5) | **<0.001** |
| **History of congestive heart failure, n (%)** | 36 (4.1) | 14 (17.3) | **<0.001** | 45 (5.1) | 5 (7.1) | 0.643 | 21 (3.2) | 29 (9.5) | **<0.001** |
| **History of peripheral vascular disease, n (%)** | 37 (4.2) | 21 (25.9) | **<0.001** | 45 (5.1) | 13 (18.6) | **<0.001** | 22 (3.4) | 36 (11.8) | **<0.001** |
| **History of cerebrovascular disease, n (%)** | 49 (5.6) | 9 (11.1) | 0.082 | 53 (6.0) | 5 (7.1) | 0.899 | 32 (4.9) | 26 (8.5) | **0.043** |
| **Dementia, n (%)** | 3 (0.3) | 6 (7.4) | **<0.001** | 7 (0.8) | 2 (2.9) | 0.281 | 1 (0.2) | 8 (2.6) | **0.001** |
| **Chronic obstructive pulmonary disease, n (%)** | 37 (4.2) | 6 (7.4) | 0.301 | 36 (4.1) | 7 (10.0) | **0.045** | 20 (3.1) | 23 (7.5) | **0.003** |
| **Connective tissue disease, n (%)** | 46 (5.3) | 2 (2.5) | 0.403 | 47 (5.3) | 1 (1.4) | 0.251 | 38 (5.9) | 10 (3.3) | 0.124 |
| **Peptic ulcer, n (%)** | 59 (6.8) | 16 (19.8) | **<0.001** | 66 (7.5) | 9 (12.9) | 0.167 | 43 (6.6) | 32 (10.5) | 0.052 |
| **Liver disease, n (%)** | 27 (3.1) | 3 (3.7) | 1.000 | 27 (3.1) | 3 (4.3) | 0.832 | 15 (2.3) | 15 (4.9) | 0.051 |
| **Diabetes mellitus, n (%)** |  |  | **<0.001** |  |  | **<0.001** |  |  | **<0.001** |
| - **no diabetes** | 662 (75.8) | 38 (46.9) |  | 666 (75.3) | 34 (48.6) |  | 515 (79.4) | 185 (60.7) |  |
| - **diabetes without complications** | 147 (16.8) | 23 (28.4) |  | 144 (16.3) | 26 (37.1) |  | 95 (14.6) | 75 (24.6) |  |
| - **diabetes with complications** | 64 (7.3) | 20 (24.7) |  | 74 (8.4) | 10 (14.3) |  | 39 (6.0) | 45 (14.8) |  |
| **Cancer, n (%)** | 88 (10.1) | 16 (19.8) | **0.013** | 94 (10.6) | 10 (14.3) | 0.457 | 56 (8.6) | 48 (15.7) | **0.002** |
| **Median Charlson Comorbidity Index (IQR)** | 4.00 [2.00, 6.00] | 8.00 [6.00, 10.00] | **<0.001** | 4.00 [2.00, 6.00] | 7.00 [5.00, 9.00] | **<0.001** | 3.00 [2.00, 6.00] | 6.00 [4.00, 8.00] | **<0.001** |
| **Standard triple combination (tacrolimus, MMF/MPA, corticosteroids)** | 391 (44.8) | 17 (21.0) | **<0.001** | 385 (43.6) | 23 (32.9) | 0.106 | 307 (47.3) | 101 (33.1) | **<0.001** |
| **Last known eGFR before COVID-19 (ml/min/1.73m^2^)** | 48.00 [34.80, 62.40] | 33.00 [22.20, 48.60] | **<0.001** | 48.00 [34.20, 62.40] | 35.40 [24.00, 48.60] | **<0.001** | 50.10 [37.20, 64.80] | 39.30 [28.20, 55.80] | **<0.001** |
| **Late contact to the transplant center, n (%)** | 188(21.5) | 40 (49.4) | **<0.001** | 193 (21.8) | 35 (50.0) | **<0.001** | 115 (17.7) | 113 (37.0) | **<0.001** |
| **Vaccination** |  |  | **0.003** |  |  | **<0.001** |  |  | **<0.001** |
| - **unvaccinated** | 432 (49.5) | 51 (63.0) |  | 429 (48.5) | 54 (77.1) |  | 281 (43.3) | 202 (66.2) |  |
| - **vaccinated with 2 doses while infected** | 137 (15.7) | 17 (21.0) |  | 146 (16.5) | 8 (11.4) |  | 113 (17.4) | 41 (13.4) |  |
| - **vaccinated with 3 doses while infected** | 304 (34.8) | 13 (16.0) |  | 309 (35.0) | 8 (11.4) |  | 255 (39.3) | 62 (20.3) |  |

**Abbreviations:** COVID-19, coronavirus disease 2019; IQR, interquartile range; PRA, panel-reactive antibodies; HLA, human leukocyte antigen; MMF, mycophenolate mofetil; MPA, mycophenolic acid; eGFR, estimated glomerular filtration rate.

**Note:** There are missing values in the Late or no contact to transplant center variable. Namely, in the 90-days mortality column, 4 (0.45%) and 4 (4.9%) missing values, in the ICU admission column, 5 (0.6%) and 3 (4.3%) missing values, in the at least moderate severity column, 0 and 8 (2.6%) missing values, respectively.

**Supplementary Table 4. Univariable predictors of late (90 days) mortality, intensive care unit admission, and at least moderately severe COVID-19.**

| **Parameter** | **Death within 90 days** | | | **Admitted to intensive care unit** | | | **At least moderate illness** | | |
| --- | --- | --- | --- | --- | --- | --- | --- | --- | --- |
|  | **OR** | **95% CI** | **p-value** | **OR** | **95% CI** | **p-value** | **OR** | **95% CI** | **p-value** |
| **Male sex** | 2.03 | 1.23, 3.51 | **0.008** | 1.41 | 0.85, 2.42 | 0.2 | 1.45 | 1.09, 1.94 | **0.011** |
| **Age at COVID** | 1.11 | 1.08, 1.14 | **<0.001** | 1.06 | 1.04, 1.08 | **<0.001** | 1.06 | 1.05, 1.07 | **<0.001** |
| **Age at COVID - category** |  |  |  |  |  |  |  |  |  |
| - **< 50 years** | — | — |  | — | — |  | — | — |  |
| - **50 – 60 years** | 4.30 | 1.30, 19.3 | **0.028** | 2.59 | 1.06, 6.93 | **0.043** | 1.81 | 1.20, 2.75 | **0.005** |
| - **60 – 70 years** | 13.1 | 4.53, 55.6 | **<0.001** | 5.55 | 2.48, 14.1 | **<0.001** | 3.53 | 2.37, 5.33 | **<0.001** |
| - **70 – 80 years** | 28.8 | 10.2, 121 | **<0.001** | 6.71 | 2.93, 17.3 | **<0.001** | 6.98 | 4.56, 10.8 | **<0.001** |
| - ***>* 80 years** | 143 | 31.2, 848 | **<0.001** | 8.66 | 1.19, 41.8 | **0.012** | 10.4 | 3.15, 40.2 | **<0.001** |
| **Years from transplantation** | 1.03 | 1.00, 1.07 | 0.060 | 1.02 | 0.99, 1.06 | 0.2 | 1.04 | 1.02, 1.06 | **<0.001** |
| **Cause of end stage kidney disease** |  |  |  |  |  |  |  |  |  |
| - **Polycystosis** | — | — |  | — | — |  | — | — |  |
| - **Glomerular disease** | 0.64 | 0.32, 1.34 | 0.2 | 0.88 | 0.44, 1.84 | 0.7 | 0.78 | 0.53, 1.16 | 0.2 |
| - **Tubulointerstitial disease** | 1.01 | 0.40, 2.42 | >0.9 | 0.71 | 0.24, 1.89 | 0.5 | 0.95 | 0.57, 1.58 | 0.9 |
| - **Diabetic kidney disease and/or vascular nephropathy** | 2.57 | 1.32, 5.26 | **0.007** | 1.77 | 0.86, 3.83 | 0.13 | 1.82 | 1.18, 2.84 | **0.008** |
| - **Others** | 1.01 | 0.37, 2.57 | >0.9 | 0.77 | 0.24, 2.15 | 0.6 | 0.73 | 0.41, 1.28 | 0.3 |
| **Lives in transplantation center vicinity** | 0.46 | 0.22, 0.88 | **0.027** | 0.49 | 0.22, 0.96 | 0.052 | 0.60 | 0.42, 0.85 | **0.005** |
| **Body mass index** | 1.08 | 1.04, 1.12 | **<0.001** | 1.07 | 1.03, 1.12 | **<0.001** | 1.05 | 1.03, 1.08 | **<0.001** |
| **Coronary artery disease** | 3.14 | 1.88, 5.14 | **<0.001** | 2.38 | 1.35, 4.08 | **0.002** | 2.07 | 1.45, 2.96 | **<0.001** |
| **Myocardial infarction** | 5.14 | 2.61, 9.68 | **<0.001** | 2.07 | 0.83, 4.51 | 0.088 | 2.86 | 1.63, 5.08 | **<0.001** |
| **Congestive heart failure** | 4.86 | 2.43, 9.28 | **<0.001** | 1.43 | 0.48, 3.42 | 0.5 | 3.14 | 1.77, 5.67 | **<0.001** |
| **Peripheral artery disease** | 7.91 | 4.31, 14.3 | **<0.001** | 4.25 | 2.10, 8.14 | **<0.001** | 3.81 | 2.22, 6.70 | **<0.001** |
| **Cerebrovascular disease** | 2.10 | 0.93, 4.26 | 0.052 | 1.21 | 0.41, 2.85 | 0.7 | 1.80 | 1.04, 3.07 | **0.032** |
| **Dementia** | 23.2 | 6.00, 112 | **<0.001** | 3.68 | 0.54, 15.6 | 0.11 | 17.5 | 3.18, 325 | **0.007** |
| **Chronic obstructive pulmonary disease** | 1.81 | 0.67, 4.13 | 0.2 | 2.62 | 1.03, 5.79 | **0.026** | 2.57 | 1.39, 4.79 | **0.003** |
| **Peptic ulcer disease** | 3.40 | 1.80, 6.12 | **<0.001** | 1.83 | 0.82, 3.68 | 0.11 | 1.65 | 1.02, 2.66 | **0.040** |
| **History of liver disease** | 1.21 | 0.28, 3.51 | 0.8 | 1.42 | 0.33, 4.16 | 0.6 | 2.19 | 1.05, 4.57 | **0.035** |
| **Diabetes mellitus** |  |  |  |  |  |  |  |  |  |
| - **no diabetes** | ref. | ref. | ref. | ref. | ref. | ref. | ref. | ref. | ref. |
| - **diabetes without complications** | 2.73 | 1.56, 4.68 | **<0.001** | 3.54 | 2.04, 6.06 | **<0.001** | 2.20 | 1.55, 3.11 | **<0.001** |
| - **diabetes with complications** | 5.44 | 2.95, 9.83 | **<0.001** | 2.65 | 1.20, 5.40 | **0.010** | 3.21 | 2.03, 5.11 | **<0.001** |
| **Cancer** | 2.20 | 1.18, 3.87 | **0.009** | 1.40 | 0.66, 2.71 | 0.3 | 1.98 | 1.31, 2.99 | **0.001** |
| **Charlson Comorbidity Index** | 1.45 | 1.35, 1.57 | **<0.001** | 1.23 | 1.14, 1.33 | **<0.001** | 1.30 | 1.23, 1.37 | **<0.001** |
| **Standard triple therapy (TAC + MMF/MPA + CS)** | 0.33 | 0.18, 0.56 | **<0.001** | 0.63 | 0.37, 1.05 | 0.084 | 0.55 | 0.41, 0.73 | **<0.001** |
| **Last known eGFR** | 0.96 | 0.95, 0.97 | **<0.001** | 0.97 | 0.95, 0.98 | **<0.001** | 0.98 | 0.97, 0.98 | **<0.001** |
| **Virus variant period** |  |  |  |  |  |  |  |  |  |
| - **Wild-type period** | — | — |  | — | — |  | — | — |  |
| - **Alpha period** | 1.34 | 0.77, 2.32 | 0.3 | 1.09 | 0.62, 1.90 | 0.8 | 1.09 | 0.76, 1.57 | 0.6 |
| - **Delta period** | 0.80 | 0.39, 1.55 | 0.5 | 0.44 | 0.18, 0.94 | **0.045** | 0.40 | 0.25, 0.62 | **<0.001** |
| - **Omicron period** | 0.25 | 0.12, 0.51 | **<0.001** | 0.12 | 0.04, 0.28 | **<0.001** | 0.22 | 0.15, 0.32 | **<0.001** |
| **Wild-type + Alpha + Delta vs. Omicron** | 0.24 | 0.11, 0.45 | **<0.001** | 0.13 | 0.05, 0.30 | **<0.001** | 0.26 | 0.18, 0.36 | **<0.001** |
| **Late contact*** | 3.27 | 2.02, 5.26 | **<0.001** | 3.36 | 2.02, 5.58 | **<0.001** | 2.54 | 1.86, 3.47 | **<0.001** |
| **Vaccination** |  |  |  |  |  |  |  |  |  |
| - **unvaccinated** | ref. | ref. | ref. | ref. | ref. | ref. | ref. | ref. | ref. |
| - **vaccinated with 2 doses while infected** | 1.05 | 0.57, 1.85 | 0.9 | 0.44 | 0.19, 0.89 | **0.033** | 0.50 | 0.34, 0.75 | **<0.001** |
| - **vaccinated with 3 doses while infected** | 0.36 | 0.19, 0.66 | **0.001** | 0.21 | 0.09, 0.41 | **<0.001** | 0.34 | 0.24, 0.47 | **<0.001** |

**Abbreviations:** OR, odds ratio; 95% CI, 95% confidence interval; TAC, tacrolimus; MMF, mycophenolate mofetil; MPA, mycophenolic acid; CS, corticosteroids; eGFR, estimated glomerular filtration rate; COVID-19, coronavirus disease 2019.

**Note:** 8 missing values.

**Supplementary Table 5. Sensitivity analyses – multivariable logistic regression models of early (30 days) mortality.**

| **Predictor** | **Model with vaccination** | | | **Model adjusted for omicron period** | | | **Model without KTRs infected during the wild-type period** | | | |
| --- | --- | --- | --- | --- | --- | --- | --- | --- | --- | --- |
|  | **OR** | **95% CI** | **p-value** | **OR** | **95% CI** | **p-value** | **OR** | **95% CI** | **p-value** |  |
| **Male sex** | 1.75 | 0.92, 3.45 | 0.095 | 1.78 | 0.95, 3.49 | 0.081 | 1.99 | 0.89, 4.81 | 0.11 |  |
| **Age at COVID-19** | 1.09 | 1.06, 1.13 | **<0.001** | 1.09 | 1.05, 1.13 | **<0.001** | 1.07 | 1.03, 1.12 | **<0.001** |  |
| **Body mass index** | 1.06 | 1.01, 1.12 | **0.030** | 1.06 | 1.01, 1.12 | **0.029** | 1.06 | 1, 1.13 | 0.064 |  |
| **Diabetes** |  |  |  |  |  |  |  |  |  |  |
| - **no diabetes** | ref. | ref. | ref. | ref. | ref. | ref. | ref. | ref. | ref. |  |
| - **diabetes without complications** | 1.23 | 0.60, 2.48 | 0.6 | 1.23 | 0.60, 2.45 | 0.6 | 1.52 | 0.63, 3.56 | 0.3 |  |
| - **diabetes with complications** | 1.57 | 0.67, 3.51 | 0.3 | 1.67 | 0.73, 3.67 | 0.2 | 1.69 | 0.59, 4.56 | 0.3 |  |
| **Standard triple therapy (TAC + MMF/MPA + CS)** | 0.75 | 0.35, 1.53 | 0.4 | 0.75 | 0.35, 1.52 | 0.4 | 0.39 | 0.14, 0.97 | 0.057 |  |
| **Latest known eGFR before COVID-19** | 0.96 | 0.94, 0.98 | **<0.001** | 0.96 | 0.95, 0.98 | **<0.001** | **0.96** | **0.94, 0.98** | **0.001** |  |
| **All waves adjustment:** |  |  |  |  |  |  |  |  |  |  |
| - **Wild-type period** | ref. | ref. | ref. | --- | --- | --- | --- | --- | --- |  |
| - **Alpha period** | 2.05 | 0.95, 4.41 | 0.066 | --- | --- | --- | ref. | ref. | ref. |  |
| - **Delta period** | 1.27 | 0.34, 4.34 | 0.7 | --- | --- | --- | 0.56 | 0.22, 1.32 | 0.2 |  |
| - **Omicron period** | 0.84 | 0.17, 3.53 | 0.8 | --- | --- | --- | 0.23 | 0.08, 0.58 | 0.003 |  |
| **Wild-type + Alpha + Delta vs. Omicron** | --- | --- | --- | 0.34 | 0.13, 0.76 | **0.013** | --- | --- | --- |  |
| **Late referral** | 2.03 | 1.06, 3.88 | **0.032** | 2.16 | 1.15, 4.00 | **0.015** | 3 | 1.36, 6.56 | **0.006** |  |
| **Vaccination:** |  |  |  |  |  |  |  |  |  |  |
| - **unvaccinated** | ref. | ref. | ref. | --- | --- | --- | --- | --- | --- |  |
| - **vaccinated with 2 doses while infected** | 0.67 | 0.25, 1.74 | 0.4 | --- | --- | --- | --- | --- | --- |  |
| - **vaccinated with 3 doses while infected** | 0.43 | 0.11, 1.86 | 0.2 | --- | --- | --- | --- | --- | --- |  |

**Abbreviations:** OR, odds ratio; 95% CI, 95% confidence interval; COVID-19, coronavirus disease 2019; TAC, tacrolimus; MMF, mycophenolate mofetil; MPA, mycophenolic acid; CS, corticosteroids; eGFR, estimated glomerular filtration rate.
